# 599. Association between Vancomycin AUC and Clinical Failure in Patients with Streptococcal Bacteremia

**DOI:** 10.1093/ofid/ofac492.651

**Published:** 2022-12-15

**Authors:** Anna C Aycock, Jessica M Smith, Kelci E Coe, Shu-Hua Wang, Erica E Reed

**Affiliations:** The Ohio State University Wexner Medical Center, columbus, Ohio; The Ohio State University Wexner Medical Center, columbus, Ohio; The Ohio State University Wexner Medical Center, columbus, Ohio; The Ohio State University Wexner Medical Center, columbus, Ohio; The Ohio State University Wexner Medical Center, columbus, Ohio

## Abstract

**Background:**

Vancomycin (VAN) is an efficacious therapy against *Streptococcus*. VAN area under the curve to minimum inhibitory concentration (AUC/MIC) is the preferred monitoring strategy for serious methicillin-resistant *S. aureus* infections but is not well elucidated for other bacterial pathogens such as *Streptococcus*.

**Methods:**

This was a retrospective cohort study evaluating adult inpatients with streptococcal bacteremia treated with VAN definitive therapy from Jan 1, 2011 to Sept 30, 2021 at a tertiary care academic medical center. VAN AUC was retrospectively calculated using Bayesian software (ClinCalc). The primary outcome was treatment failure, defined as a composite of recurrent or persistent streptococcal bacteremia, 60-day all-cause readmission, or 60-day all-cause mortality. Secondary outcomes included time to bacteremia clearance, hospital length of stay (LOS), and nephrotoxicity. Data collected included demographics; comorbidities; severity of illness; streptococcal species and source; VAN initial trough and duration; and clinical outcomes. Classification and regression tree analysis (CART) was conducted to identify the AUC threshold predictive of clinical failure. Wilcoxon rank sum, Chi Square, or Fisher’s exact tests were utilized as appropriate to compare groups stratified by the CART-identified AUC threshold.

**Results:**

Forty-six patients met inclusion criteria during the study timeframe. Eleven patients had a VAN AUC < 329 of which 8 (73%) experienced clinical failure, while 35 patients had a VAN AUC > 329 of which 12 (34%) experienced clinical failure (p=0.04). No significant differences in baseline or clinical characteristics were identified between groups. Median VAN initial trough was higher in the VAN AUC > 329 group (13.2 vs 6.2, p< 0.001). Median hospital LOS was longer in the VAN AUC > 329 group (15 vs 8 days, p=0.05) while median time to bacteremia clearance (29 vs 25 hrs, p=0.15) and nephrotoxicity incidence (13% vs 4%, p=1) were not significantly different.

**Conclusion:**

Vancomycin AUC < 329 was predictive of clinical failure in patients with streptococcal bacteremia. Larger studies are needed before VAN AUC monitoring can be recommended for implementation in the management of streptococcal bacteremia.

**Disclosures:**

**All Authors**: No reported disclosures.

